# Brain Metastases from Gynecologic Malignancies

**DOI:** 10.3390/medicina58040548

**Published:** 2022-04-15

**Authors:** Georgia Karpathiou, Florian Camy, Céline Chauleur, Maroa Dridi, Pierre Dal Col, Michel Peoc’h

**Affiliations:** 1 Pathology Department, University Hospital of Saint-Etienne, 42055 Saint-Etienne, France; florian.camy@chu-st-etienne.fr (F.C.); maroa.dridi@etu.univ-st-etienne.fr (M.D.); pierre.dal-col@chu-st-etienne.fr (P.D.C.); michel.peoch@chu-st-etienne.fr (M.P.); 2 Gynecology and Obstetrics Department, University Hospital of Saint-Etienne, 42055 Saint-Etienne, France; celine.chauleur@chu-st-etienne.fr

**Keywords:** ovarian cancer, endometrial cancer, brain metastasis

## Abstract

*Background and Objectives*: To present a series of brain metastases from gynecologic primaries and provide a summary of the relevant literature. *Materials and Methods*: We retrospectively review 18 patients with histologically confirmed brain metastases from gynecologic primaries and summarize the largest series of relative reports. *Results*: Six brain metastases were of endometrial primary and 12 of ovarian primary. In 3 cases (16.7%), diagnosis of brain metastases was made at presentation of the gynecologic primary; in the others, median time to development of brain metastasis was 34 (range, 6–115) months. Median survival after brain metastasis diagnosis was 5 (range, 1–89) months. Favorable prognostic factors were better performance status (*p* = 0.04) and, marginally, smaller metastasis size (*p* = 0.06). No differences in brain metastases between endometrial and ovarian primaries were found, except for the time interval from primary to brain metastases diagnosis, which was shorter for endometrial tumors (*p* = 0.05). A comprehensive summary of previous studies is provided. *Conclusions*: Performance status and smaller brain metastases size are good prognostic factors. Endometrial cancer brain metastases develop earlier than ovarian cancer brain metastases.

## 1. Introduction

Endometrial and ovarian cancer consist almost 80% of all gynecologic malignancies [1], the former showing a generally good prognosis with a 5-year survival of about 80% for earlier stages [2] and the latter a 5-year overall survival of almost 40% [3]. Endometrial cancer metastasizes more frequently to the lung, while ovarian cancer propagates intra-peritoneally with frequent liver metastases [4]. Brain metastasis from any gynecologic cancer is very unusual, except for trophoblastic disease [5]. In large retrospective Surveillance, Epidemiology, and End Results (SEER) database study, ovarian cancer and uterine cancer patients with metastatic disease at presentation, including lung, liver, bone and brain, showed brain metastases in 1% and 3% of the cases, respectively [4]. Moreover, this metastatic site was significantly associated with worse prognosis, showing a 10% 5-year survival rate [4]. However, an augmented diagnosis of brain metastases has been observed [6], probably associated with prolonged survival after modern treatments and with new imaging techniques. Thus, any data regarding this rare phenomenon will improve our knowledge.

In this report, we aim to describe the clinicopathological features of a brain metastases case series and to present a summary of the available literature.

## 2. Materials and Methods

This is a retrospective single-center study of brain metastases histologically confirmed after biopsy/resection of the brain metastasis. Primary tumor origin, patient’s age and performance status at brain metastasis diagnosis, brain metastasis histological type, brain metastasis localization, size, multiplicity, and interval from primary cancer diagnosis, treatment and survival after brain metastasis diagnosis were recorded. Comité d’Ethique du CHU de Saint-Etienne and Commission recherche de Terre d’éthique approved the study (IRBN502017/CHUSTE, approval date: 28 September 2017).

Statistical analysis was performed using the StatView(Version 5) software (Abacus Concepts, Berkeley CA, USA). We used factorial analysis of variances (ANOVA) and the Mann-Whitney U test to consider the effect of at least one factor on a continuous parameter and the Fisher’s exact test to explore any relationship between two groups for categorical data. For all analyses, statistical significance was set at a *p* value of <0.05. Survival probability was estimated by Kaplan-Meier analysis with log-rank product limit estimation.

## 3. Results

Eighteen patients (Table 1), 6 (33.3%) with endometrial cancer and 12 (66.7%) with ovarian cancer were identified. All ovarian tumors were high grade serous carcinomas; 5 endometrial tumors were grade 3 endometrioid carcinomas and one was grade 2 endometrioid carcinoma. Median age at brain metastasis diagnosis was 63.5 years (35–82). Performance status was 0–1 for 10 patients (55.5%) and >1 for 8 (44.5%). All but one cases were symptomatic. Three cases were identified at the same time with the primary tumor (16.7%). For the rest, the median interval time from primary tumor to brain metastasis was 34 months (6–115). Brain metastases were multiple in two cases (11.1%). Eleven cases were cerebral (61.1%), while 7 (38.9%) cerebellar. The median size of the brain metastasis was 30 mm (11–42) and this cut off was used to group the cases for further analysis. 16 cases were complete excisions, while two brain biopsies. Adjuvant treatment was administrated in 12 (75%) patients, 9 of these with radiotherapy only. Median overall survival after brain metastasis diagnosis was 5 (1–89) months.

Most endometrial primaries (*n* = 5, 83.3%) and half ovarian primaries presented with cerebral brain metastases, but the difference (Table 2) was not statistically significant (*p* = 0.3). The two cases of multiple brain metastases were ovarian primaries, while all endometrial cases were solitary lesions (*p* = 0.5). The two primaries did not differ in brain metastasis size (*p* = 0.6). Most brain metastases (*n* = 5, 83.3%) from endometrial primaries were diagnosed at the age of >60 years, while half of ovarian cases were diagnosed in this age group (*p* = 0.3). The interval time from primary tumor to brain metastases diagnosis differed between the two primaries, since metastases from endometrial primaries were diagnosed at a mean of 27.8 months, while from ovarian at 53.5 months (*p* = 0.05). There was no difference at the performance status of the patients between the two groups (*p* = 0.4).

**Figure 1:**
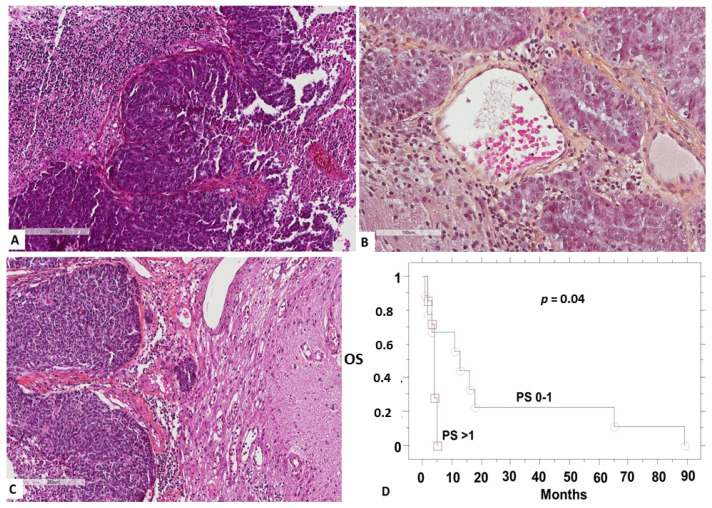
(**A**) Ovarian high grade serous carcinoma infiltrating cerebellar tissue (left). Papillary structures and necrosis seen at the right. (**B**) Grade 2 endometrioid adenocarcinoma infiltrating glial tissue (left). (**C**) Grade 3 endometrioid adenocarcinoma infiltrating glial tissue (right). (**D**) Overall survival (OS) according to performance status.

Microscopic examination of brain metastases showed that all endometrial brain metastases were endometrioid adenocarcinomas (5 grade 3, one grade 2) and all ovarian brain metastases were high grade serous carcinomas (Figure 1).

Survival analysis showed no differences between the two primaries (*p* = 0.5), the localization of brain metastasis (*p* = 0.5), their multiplicity (*p* = 0.2), or the adjuvant treatment (*p* = 0.3). There was a strong trend (*p* = 0.06) for better overall survival in cases with size less than 30 mm. Patients with good performance status also showed improved overall survival after brain metastasis diagnosis (*p* = 0.04, Figure 1).

## 4. Discussion

In the current series, median overall survival after brain metastases diagnosis was 5 months and good performance status and smaller metastasis size were favorable prognostic factors. Despite these findings should be interpreted with caution given the limited number of patients, previous studies (Table 3) similarly showed a median overall survival ranging from 5 to 12 months, and the most frequently reported favorable prognostic factors were single metastases and good performance status, probably reflecting surgery eligibility and complete brain metastasis resection. However, treatment modalities vary considerably in these studies, as brain metastasis diagnosis in their vast majority was based in imaging, while surgery has been part of the treatment in a small portion of patients (Table 3). In the current series, all but 2 patients have been surgically treated, and we show that in this context performance status and smaller size also predict better survival.

Furthermore, most of these studies do not report in brain metastasis histology; studies which report in histology are based in the primary tumor’s reports (Table 3). The current series, in comparison to previous studies which comprised various carcinomas, carcinosarcomas, sarcomas, germ cell and gestational tumors, is homogenous also in terms of histologic type, as all endometrial tumors were endometrioid carcinomas, most grade 3, and all ovarian tumors were high grade serous carcinomas.

Given the rarity of brain metastases from gynecologic malignancies, endometrial as well as ovarian tumors are usually included in the studies. However, these are biologically different tumors, and it would be interesting to know if there are any differences regarding their brain metastases. We found that endometrial and ovarian brain metastases differed only in the time interval between primary cancer and metastasis diagnosis, as it was shorter for endometrial cancer. This time interval in other studies ranged from 5 to 28 months for all cancers included, but only three studies provide details regarding their primaries with contradictory results. Zhang et al. showed an interval of 22.8 months for both uterine corpus and ovarian primaries [5], Takeshita et al. showed 22- and 28-months interval for uterine and ovarian tumors, respectively [7], while Kim et al. showed 27.8- and 21.6-months interval for uterine and ovarian tumors, respectively [8]. In these two latter studies, no statistical comparison between primaries regarding this interval time is provided. At the same time, in the study of Takeshita et al. [7], ovarian primary was related with better survival, but this significance was lost when the treatment-free interval time was incorporated in the analysis, probably reflecting the importance of primary-metastasis interval. On the other hand, the study of Kim et al. [8], showed that uterine and not ovarian primary cancer was associated with better survival after brain metastasis diagnosis. This comes in contrast also to another study, that of Divine et al. [9], showing that ovarian primary is associated with better survival. Growdon et al. concluded that serous histology vs. other tumors, but not ovarian vs. endometrial was associated with better survival [10]. These contradictory results could in part be associated with the time interval between primary and brain metastasis diagnosis, which as we show here is shorter for uterine than ovarian tumors. Our findings would be in line with the two studies of endometrial-only brain metastases, where a relatively short interval time was also seen [11,12]. The reasons for this shorter interval for endometrial vs. ovarian primaries are not clear, but a correlation with the most often hematogenous spread of uterine cancer could be hypothesized.

Regarding other brain metastases characteristics, the current and the previous series showed that most cases are of ovarian primary, followed by endometrial cancer. The reported incidence of brain metastases among patients with gynecologic cancer is 0.6–2.7%. Patients are usually diagnosed at the 6th–7th decade. Most brain metastases (46.1–66.7%) are cerebral. Brain metastases are multiple in 37.7–84.6% of the reported cases, while this was lower in our series (11.1%), probably associated with the type of patients included as previously mentioned. When the information is available, brain metastasis is diagnosed synchronously with the primary in 4–25% of the cases, like in our series.

**Table 3:** Main findings of the largest gynecologic metastases brain metastases series.

Ref.	*n*	Primary Tumor (*n*) ^#^			Histology of Primary Tumor	Incidence of Brain Metastasis *	Synchronous Lesions	Multiple Lesions	Localization	Age at Brain Metastasis	Time to Brain Metastasis (Months)	Survival after Brain Metastasis	Surgery Included in Treatment
		EN	OV	CER									
Zhang [4]	42	24	9	5	Ovary: 88.8% serous Uterine corpus: Endometrioid 45.8% (4 G1-G2, 7 G3) Serous 16.6% Carcinosarcoma 12.5%	NA	NA	67%	Most frontal	61.2 (median)	20.7 (0–107) 22.8 for both uterine corpus and ovarian	NA	Treatment NA
Uccella [12]	18	18	0	0	Endometrioid 66.7% (G1 = 2, G2 = 2, G3 = 8) Adenosquamous 5.6% Serous 16.7% Undifferentiated 11.1%	0.8%	11%	50%	Supratentorial 66.7% Infratentorial 11.1% Both 22.2%	64 (42–82)	5 (1–57)	57 (7–118) months for 6 patients with single meta and no extracranial lesions 4 (0–28) months for the other 12 patients	44.4%
Takeshita [7]	47	12	17	18	Uterine corpus: Endometrioid 50% Serous 2.5% Carcinosarcoma 1.7% Others + Ovary: Serous 65% Clear cell 18.8% Undifferentiated 5.9% Others +	1.7%	25%	49%	NA	62	26 (0–194) Uterine corpus: 22 Ovary: 28	5 (4–5 months) months The 6-, 12- and 24-month survival rates: 44.0%, 22.0% and 16.5%, respectively. Poor survival: extracranial metastasis, performance status 3–4, treatment-free interval of <6 months and no anti-cancer treatment for brain metastasis	24.4%
Ratner [13]	24	0	23	0	Serous 70.9% Endometrioid 12.5% Undifferentiated 16.6%	1%	NA	70.8%	Cerebral 62.5% Cerebellar 16.6%	56 (37–78)	23 (1–74)	8.5 months (1–97) with a 42% 1-year survival and 16% 2-year survival. Patients with single lesions had longer survival than patients with multiple lesions	16.6%
Rades [14]	42	11	22	9	NA	NA	NA	81% ≥4 lesions	NA	50% ≥60 years	NA	5 months Improved survival was associated with performance status ≥70, <4 brain metastases, and lack of extracranial metastases	No surgery
Marchetti [6]	174	0	174	0	77.6% serous 7.5% endometrioid 5.2% clear cell 6.3% undifferentiated Others	NA	NA	57.6%	NA	60 (35–88)	26 (2–129)	12 (9.6–14.3) Poor overall survival: multiple brain metastasis, extracranial disease, age and monotherapy	35.6%
Kim [15]	13	0	13	0	Serous 69.2% Others +	2.7%	NA	84.6%	Supratentorial 46.1% Cerebellar 7.8% Both 46.1%	52 (31–73)	28 (13–99)	7 months Prognostic factors: Performance status >70, primary control, solitary brain metastases, gamma-knife radiosurgery	7.6%
Kim [8]	61	19	32	10	Uterine corpus: 17 carcinomas Ovarian: 23 epithelial neoplasms No further details		11.5%	37.7%	Supratentorial 65.9% Infratentorial 31.8% Leptomeninges 2.3%	54.7 (23–80)	25.4 (0–84.3) Uterine corpus: 27.8 Ovary: 21.6, but no direct comparison	Type of primary cancer (uterine), performance status, status of primary cancer, recursive partitioning analysis class, and combined therapies, were significantly related to the overall survival	32.7%
Growdon [10]	47	12	30	3	Serous 26 Others 21 Not further described	0.9%	NA		Cerebral 38.3% Cerebellar 25.5% Both 36.2%	12 >60	>24 months for 22 patients No comparison between primaries	7.5 median (9 d–64 m), 40% 1-year survival Extracranial disease predicted decreased survival, while use of any chemotherapy predicted longer survival. Also, serous histology predicted longer survival, but ovarian vs. endometrial primary showed no difference	38.3%
Gien [11]	8	8	0	0	Endometrioid 37.5% (all G3) Serous 25% Clear cell 12.5% Carcinosarcoma 12.5% Adenosquamous 12.5%	0.6%	25%	50%	Cerebellar 25% cerebral 50% both 25%	66 (48–82)	8.5 (2–40)	3.5 months	No surgery
Divine [9]	100	32	49	19	Serous 38% Endometrioid 19% Others +		4%	NA	NA	59.4 mean (32–85)	33.6 ± 30.4 (0–164.8) No comparison between primaries	Primary ovarian disease, CA-125 at brain metastasis diagnosis, and isolated metastases were all associated with longer survival.	
D’Andrea [16]	11	0	11	0	All serous		None	None, all solitary	9 cerebral 2 cerebellar	60.3 med	21 median	Mean 28 months	All surgery
Owaga [17]	18	NA	7	NA	7 squamous Others not defined	0.7%		13		55 (42–74)	16 (0.78) No comparison between primaries	Median 4.1 months 11% 1 y and 2 y survival Prognostic factors: Performance status, extracranial disease, number of brain metastases	33.3%
Anupol [18]	15	0	15	0	Serous 93.3% Carcinosarcoma 6.7%	1.4%		7			22 median	Median 6 months	33.3%
Mahmud-Ahmed [19]	25	10	9	6	Adenocarcinoma (no further details) 80% Adenosquamous 8% Squamous 12%				19 cerebral 2 cerebellar 4 both	46 (37–78)	12.7 (0.2–106.5) No comparison between primaries	Median 7.3 months Better with single brain metastasis	40%

*: Estimated incidence of brain metastasis among patients diagnosed with a gynecological, endometrial, or ovarian cancer at the same period. +: Several other subtypes. #: Other types not included in the 3 mentioned origins: Zhang gestational, Yano peritoneal, Ratner peritoneal. EN endometrial, OV ovarian, CER cervical. NA not available

## 5. Conclusions

To conclude, brain metastases from gynecologic malignancies is a rare phenomenon, and the data of the current series and previous series should be interpreted with caution given the limited number of patients studied. Performance status and brain metastases size have prognostic significance for surgically treated patients. Endometrial cancer brain metastases develop earlier than ovarian cancer brain metastases.

## Figures and Tables

**Table 1:** Patients’ characteristics.

Primary tumor	
Ovarian carcinoma	12 (66.7%)
Endometrial carcinoma	6 (33.3%)
Age at brain metastasis diagnosis	
Mean	61.7 ± 12.7
Median (range)	63.5, (35–82)
Performance status	
0–1	10
>1	8
Size (mm)	
Mean	29.2 ± 8.4
Median (range)	30 (11–42)
Interval from primary diagnosis (months)	
Mean	42.9 ± 32.9
Median (range)	34 (6–115)
Survival after metastasis diagnosis (months)	
Mean	17.2 ± 26.2
Median (range)	5 (1–89)
Brain metastasis localization	
Cerebral	11 (61.1%)
Frontal	5 (27.8%)
Temporal	4 (22.2%)
Parietal	2 (11.1%)
Cerebellar	7 (38.9%)
Multiple	
No	16 (88.9%)
Yes	2 (11.1%)
Synchronous metastases	
Yes	3 (16.7%), all ovarian
No	15 (83.3%)

**Table 2:** Comparison between the two primaries.

	Ovarian Carcinoma *n* = 12	Endometrial Carcinoma *n* = 6	*p*
Age at metastasis diagnosis (>60)			
Yes	6	5	0.3
No	6	1	
Performance status			
0–1	7	3	0.4
>1	5	3	
Size	29.1	29.3	0.6
Interval from primary diagnosis	27.8	53.5	0.05
Brain metastasis localization			
Cerebral	6	5	0.3
Cerebellar	6	1	
Multiple brain metastases			
No	10	6	0.5
Yes	2	0	
Synchronous metastases			
Yes	3	0	0.5
No	9	6	

## Data Availability

Data are available upon reasonable request.
